# X-ray Diffraction Analysis to Explore Molecular Traces of Eccentric Contraction on Rat Skeletal Muscle Parallelly Evaluated with Signal Protein Phosphorylation Levels

**DOI:** 10.3390/ijms222312644

**Published:** 2021-11-23

**Authors:** Kazuhiro Hirano, Hideki Yamauchi, Naoya Nakahara, Kazuo Kinoshita, Maki Yamaguchi, Shigeru Takemori

**Affiliations:** 1 Department of Molecular Physiology, Division of Physical Fitness, The Jikei University School of Medicine, Tokyo 182-8570, Japan; yamauchi@jikei.ac.jp; 2 Department of Molecular Physiology, The Jikei University School of Medicine, Tokyo 105-8461, Japan; nkhr@jikei.ac.jp (N.N.); maki@jikei.ac.jp (M.Y.); sml@jikei.ac.jp (S.T.); 3 Rehabilitation Medicine, The Jikei University School of Medicine Kashiwa Hospital, Chiba 277-8567, Japan; kinop@jikei.ac.jp

**Keywords:** eccentric contraction, X-ray diffraction, FoxO, mTOR, MAPK, troponin, myosin

## Abstract

We performed X-ray diffraction analyses on rat plantaris muscle to determine if there are strain-specific structural changes at the molecular level after eccentric contraction (ECC). ECC was elicited in situ by supramaximal electrical stimulation through the tibial nerve. One hour after a series of ECC sessions, the structural changes that remained in the sarcomere were evaluated using X-ray diffraction. Proteins involved in cell signaling pathways in the muscle were also examined. ECC elicited by 100, 75, and 50 Hz stimulation respectively developed peak tension of 1.34, 1.12 and 0.79 times the isometric maximal tetanus tension. The series of ECC sessions phosphorylated the forkhead box O proteins (FoxO) in a tension-time integral-dependent manner, as well as phosphorylated the mitogen-activated protein kinases (MAPK) and a protein in the mammalian target of rapamycin (mTOR) pathway in a maximal tension dependent manner. Compared to isometric contractions, ECC was more efficient in phosphorylating the signaling proteins. X-ray diffraction revealed that the myofilament lattice was preserved even after intense ECC stimulation at 100 Hz. Additionally, ECC < 75 Hz preserved the molecular alignment of myoproteins along the myofilaments, while 75-Hz stimulation induced a slight but significant decrease in the intensity of meridional troponin reflection at 1/38 nm^−1^, and of myosin reflection at 1/14.4 nm^−1^. These two reflections demonstrated no appreciable decrease with triple repetitions of the standard series of ECC sessions at 50 Hz, suggesting that the intensity decrease depended on the instantaneous maximal tension development rather than the total load of contraction, and was more likely linked with the phosphorylation of MAPK and mTOR signaling proteins.

## 1. Introduction

Eccentric contraction (ECC) is a contraction mode of skeletal muscles wherein the force-generating sarcomeres elongate to induce an augmented force. In the field of physical fitness and sports medicine, ECC has received special attention because it can have dual effects on skeletal muscle: injury [1,2,3,4] or strengthening [5,6,7]. Strenuous ECC readily injures muscles. For instance, downhill running in rats has been shown to injure the plantaris muscles by attenuating contractility, which is accompanied by an inflammatory response that occurs within a few days, followed by the recovery of muscle contractility with fiber remodeling in a few weeks [8]. Augmented force stacked from each sarcomere impairs various possible extra-sarcomeric sites along the force transmitting pathway, such as the costamere, sarcolemma, neuromuscular junction, musculotendinous junction, and fascia.

Muscle strengthening effects, on the other hand, are suggested to be specifically efficient in ECC compared with other contraction modes [9]. Even mild ECC has been shown to strengthen muscles without inducing muscle injury at the cellular level [5,7,10]. Some subcellular sites that are strained in ECC are responsible for triggering muscle strengthening effects. Probable candidates for the responsible sites reside within sarcomere structures, where a limited number of force-bearing cross-bridges are forced to extend, imposing an uneven intense strain on the thick and thin filaments. Forces stacked from each sarcomere during mild ECC may cause extra-sarcomeric strain at an intensity that is at the most comparable to that induced by an intense contraction of other modes.

In the present study, we aimed to explore ECC-specific traces of strain in sarcomere structures using X-ray diffraction while monitoring forkhead box O (FoxO), mitogen-activated protein kinases (MAPK), and mammalian target of rapamycin (mTOR)-related signal proteins. A series of ECC sessions of intensity near the threshold for the phosphorylation of signal proteins were found to result in slight but significant traces of local strain on thick and thin filaments, candidates for the sites responsible for the mediation of muscle strengthening effects of mild ECC.

## 2. Results

### 2.1. Contractile Performance during ECC Sessions

**Figure 1:**
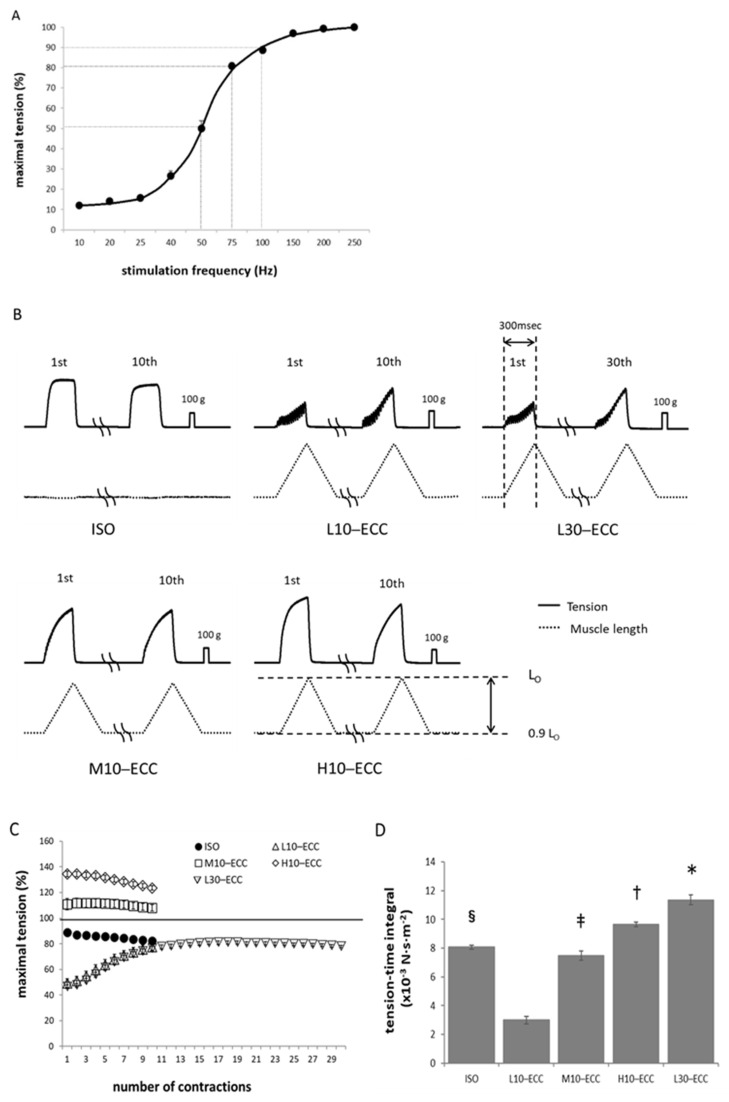
Contractile response of plantaris muscle. (**A**) Isometric tetanus tension (relative to maximal) vs. frequency. Average of eight muscles. (**B**) Representative traces of tension (solid) and muscle length (dotted) during the first and last contraction sessions. (**C**) Peak tension during the session series. Invisible error bars are smaller than the symbol size. (**D**) Tension-time integral of the session series. * *p* < 0.01 vs. ISO, L10–ECC, M10–ECC, H10–ECC; ^†^ *p* < 0.01 vs. ISO, L10–ECC, M10–ECC; ^‡^ *p* < 0.01 vs. L10–ECC; ^§^ *p* < 0.01 vs. L10–ECC.

Six groups of the rat plantaris muscles received a series of contraction sessions of different strengths and durations in situ (Table 1); H10–ECC (high), M10–ECC (moderate), and L10–ECC (low) received 10 ECC sessions at 100-, 75-, and 50-Hz stimulation, respectively. The isometric contraction group (ISO) received 10 sessions of isometric contraction at 100-Hz stimulation, and the L30–ECC group received 30 ECC sessions at 50-Hz stimulation for comparison. The control group (CON) received no contraction sessions (for details, see the Methods section). The H10–ECC, M10–ECC, and L10–ECC groups demonstrated peak tensions of 1.34 ± 0.02 (*n* = 12), 1.12 ± 0.04 (*n* = 13), and 0.79 ± 0.02 (*n* = 26) times that of the isometric maximal tetanus tension at L_0_ (Figure 1B,C), respectively. Tension during the session series generally showed a slight decrease with repetition of contraction, and a higher tension in H10–ECC and M10–ECC than in ISO was maintained. L10–ECC and the initial 10 sessions of L30–ECC showed a gradual tension increase to a level comparable to that of ISO. The last 10 sessions of L30–ECC showed a slight decrease. The tension-time integral during the session series was the smallest in L10–ECC and largest in L30–ECC (Figure 1D).

In the present study, we used the maximal tension encountered during the session series as an index of the instantaneous intensity of each contraction series, and the tension-time integral as an index of the overall mechanical load of the series. Note that the maximal tension of L10–ECC, L30–ECC, and M10–ECC was comparable to the maximal isometric tetanus tension at 200–250 Hz (Figure 1C).

### 2.2. Contractility after the Session Series

**Figure 2:**
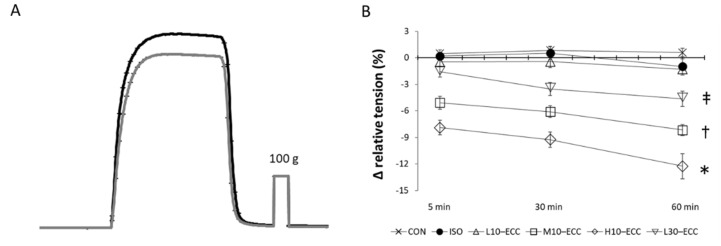
Contractility after the contraction sessions. (**A**) Averaged traces of maximal isometric tension development of H10–ECC before (black) and 1 h after (gray) the contraction sessions (*n* = 8). Invisible error bars are smaller than the symbol size. (**B**) Maximal isometric tension after the contraction sessions expressed as % difference from the tension before the sessions. * *p* < 0.01 vs. CON, ISO, L10–ECC, M10–ECC, L30–ECC; ^†^ *p* < 0.01 vs. CON, ISO, L10–ECC; ^‡^ *p* < 0.01 vs. CON.

Figure 2 shows the results of contractility evaluation after the contraction sessions. For 1 h after the contraction sessions, ISO showed sustained maximal tension without a sign of secondarily evoked changes such as denervation effects. Compared with the sustained level of ISO, H10–ECC showed a marked decline in maximal isometric tension, indicating considerable deterioration in muscle function. M10–ECC and L30–ECC showed a continuous gradual decline in maximal isometric tension, representing the progress of considerable aftereffects of the contraction sessions.

### 2.3. Signal Proteins

To ensure that the present ECC sessions were intense enough to induce muscle strengthening, we examined the phosphorylation levels of FoxO, MAPK, and mTOR related proteins in parallel with the structural evaluation by X-ray diffraction. Here, the phosphorylation level was expressed as the ratio of phosphorylated protein to total protein on the basis of band densities separately stained with respective primary antibodies (for details, see the Methods section). Both biochemical and structural evaluations were performed on muscle samples dissected 1 h after the contraction sessions, as the steadily continuous changes in contractility shown in Figure 2 indicates progress of aftereffects of sessions without any secondarily evoked effects at the given point in time.

**Figure 3:**
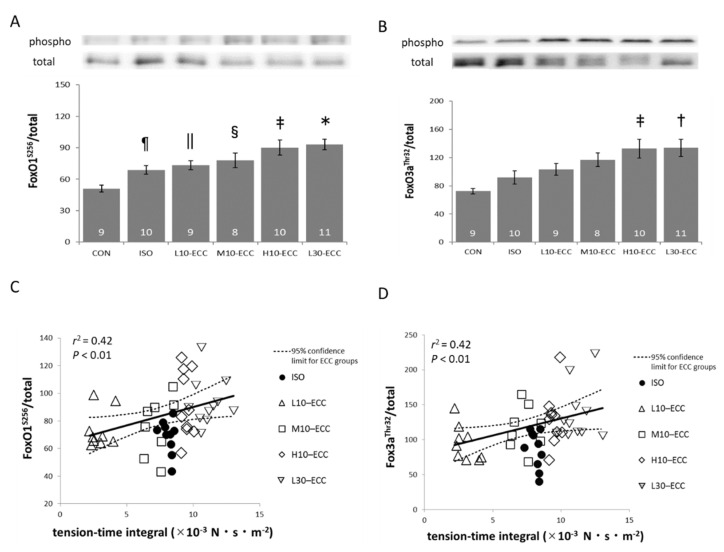
Western blot analyses of FoxO signals. (**A**,**B**) Band images and phosphorylation levels of FoxO1 (**A**) and FoxO3a (**B**). Inside the bars are the number of samples. (**C**,**D**) Respective scatter plots showing the correlations with the tension-time integral. Linear regression indicated that the probability of obtaining the data set of ISO as a part of the data set of ECC group was less than 0.01 for both proteins. * *p* < 0.01 vs. CON, ISO; ^†^ *p* < 0.01 vs. CON; *p* < 0.05 vs. ISO; ^‡^ *p* < 0.01 vs. CON; ^§^ *p* < 0.05 vs. CON; ^||^ *p* < 0.05 vs. CON; ^¶^ *p* < 0.05 vs. CON.

The phosphorylation levels of FoxO1 and FoxO3 in the muscles after ECC sessions increased most prominently in L30–ECC, which exhibited the largest tension-time integral in the present study (Figure 3A,B). Therefore, we integrated the data sets of H10–ECC, M10–ECC, L10–ECC, and L30–ECC into the merged data set, “the ECC group”, to analyze the correlation of FoxO phosphorylation levels with the tension-time integral. Significant correlations (*p* < 0.01) were found for both FoxO1 and FoxO3 (Figure 3C,D). Since total protein of FoxO1 and FoxO3 seemed to decrease after the contraction sessions (Figure 3A,B; cumulated data not shown), the increased phosphorylation level after the contraction sessions indicated marked decrease in the unphosphorylated fraction of the proteins.

**Figure 4:**
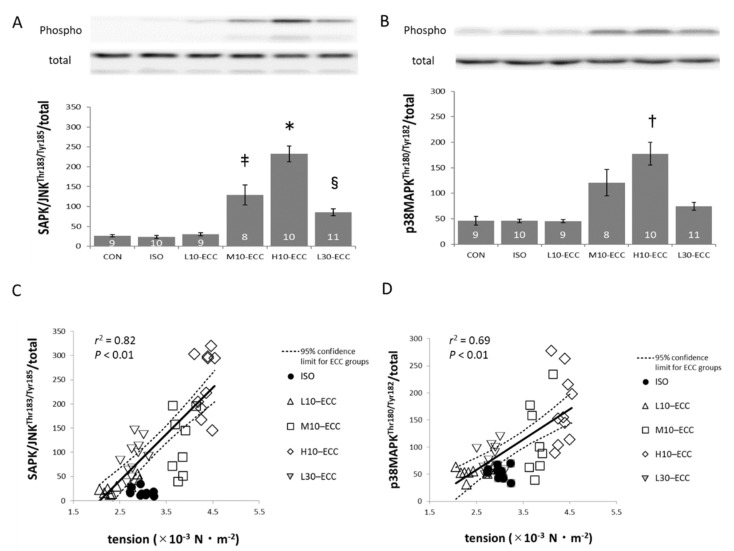
Western blot analysis of MAPK signals. (**A**,**B**) Band images and phosphorylation levels of SAPK/JNK (**A**) and p38MAPK (**B**). Inside the bars are the numbers of samples. (**C**,**D**) Respective scatter plots showing the correlations with the maximal tension exhibited during the series of contraction sessions. Linear regression indicated that the probability of obtaining the data set of ISO as a part of the data set of ECC group was less than 0.01 for both proteins. * *p* < 0.01 vs. CON, ISO, L10–ECC, L30–ECC; *p* < 0.05 vs. M10–ECC; ^†^ *p* < 0.01 vs. CON, ISO, L10–ECC, L30–ECC; ^‡^ *p* < 0.05 vs. CON, ISO, L10–ECC; ^§^ *p* < 0.05 vs. CON, ISO, L10–ECC.

On the other hand, two key proteins of the MAPK family, SAPK/JNK and p38MAPK, showed marked phosphorylation most prominently in H10–ECC, which exhibited the largest maximal tension in the present study (Figure 4A,B). The integrated data set of the ECC group showed that phosphorylation levels of both SAPK/JNK and p38MAPK were significantly correlated with the maximal tension encountered during the contraction sessions (*p* < 0.01, Figure 4C,D). Linear regression lines for the correlations intercept the axis of maximal tension at positive values slightly below the level of L10–ECC. As for ERK of the MAPK family, no appreciable difference was found among the groups (data not shown).

**Figure 5:**
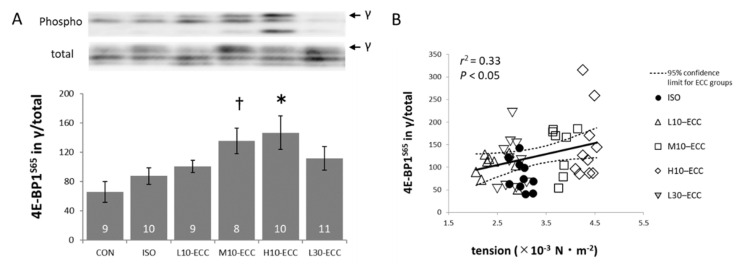
Western blot analysis of an mTOR related signal. (**A**) Band images and the level of S65 phospho-form of γ-fraction in total 4E-BP1. Inside the bars are the numbers of samples. (**B**) Scatter plot with the maximal tension exhibited during the series of contraction sessions. Linear regression indicated that the probability of obtaining the data set of ISO as a part of the data set of ECC group was less than 0.05. Similar correlation was found for the abundance of γ-fraction in total 4E-BP1 with the maximal tension in ECC group (*r*^2^ = 0.34, *p* < 0.05). * *p* < 0.01 vs. CON; *p* < 0.05 vs. ISO; ^†^ *p* < 0.05 vs. CON.

One of the proteins of the mTOR pathway, 4E-BP1, showed an increase in the S65 phospho-form in its γ-fraction most prominently in H10–ECC, which exhibited the largest maximal tension in the present study. In the integrated ECC group, a significant correlation (*p* < 0.05) was found with the maximal tension for the abundance of the S65 phospho-form of the γ-fraction as well as the whole γ-fraction in total 4E-BP1 (Figure 5).

Another protein of the mTOR pathway, p70S6K, showed a significant increase in phosphorylation level most prominently in H10–ECC, but the correlation with maximal tension in the ECC group was insignificant (data not shown).

Figure 3, Figure 4 and Figure 5 show that the ISO dataset is predominantly located below the regression lines for the data set of the integrated ECC group. This clearly indicates that ECC phosphorylates FoxO1 and FoxO3 proteins more efficiently for a given tension-time integral, and SAPK/JNK, p38MAPK, and 4E-BP1 more efficiently for a given maximal tension during the contraction sessions.

### 2.4. Myofilament Lattice and Passive Tension at Resting Condition

**Figure 6:**
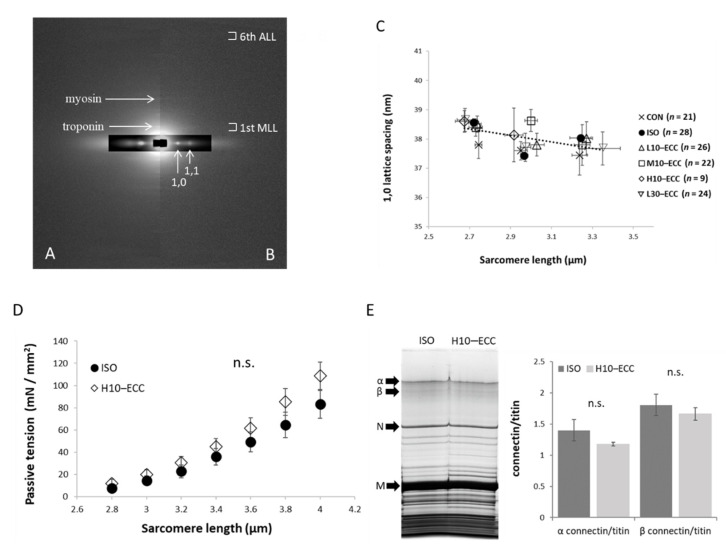
Analyses of resting sarcomere (**A**,**B**). Representative X-ray diffraction patterns from ISO (left half, **A**) and H10–ECC (right half, **B**) at resting condition. 1st MLL, 1st-order myosin layer line; 6th ALL, 6th-order actin layer line; myosin, 3rd-order meridional myosin reflection at 1/14.3 nm^−1^; troponin, troponin reflection at 1/38 nm^−1^. (**C**) 1,0 lattice spacing vs. sarcomere length. No data point for H10–ECC at sarcomere length > 3.1 μm. (**D**) Passive tension vs. sarcomere length of 14 mechanically skinned fibers per each group of ISO and H10–ECC. (**E**) Electrophoretic analysis of connectin/titin. α, α connectin/titin; β, β connectin/titin; N, nebulin; M, myosin heavy chain. *n* = 3 per group. Ordinate: band density ratio of connectin/titin relative to nebulin.

To obtain structural information of the muscles after the contraction sessions, we first obtained X-ray diffraction patterns under the resting conditions where myosin heads are detached from actin (Figure 6). Equatorial 1,0 and 1,1 reflections were readily recognized in all the groups, while meridional reflections and layer lines originating from molecular arrangements of thin and thick filaments were largely attenuated in H10–ECC (Figure 6A,B). The well-preserved equatorial reflections indicated that the lattice alignment of the myofilaments was preserved in all groups. The spacing of the equatorial reflections showed no appreciable effect of ECC sessions on the spacing of the myofilaments (Figure 6C). The intensity ratio of 1,1 reflection to that of 1,0 reflection (1,1/1,0 intensity ratio) showed no appreciable difference among the groups: CON 0.50 ± 0.04 (*n* = 16), ISO 0.43 ± 0.05 (*n* = 23), L10–ECC 0.43 ± 0.04 (*n* = 22), M10–ECC 0.40 ± 0.04 (*n* = 22), H10–ECC 0.53 ± 0.03 (*n* = 8), and L30–ECC 0.51 ± 0.06 (*n* = 21), indicating that the distribution of myosin heads around the shaft of thick filaments were preserved in all groups at the resting condition.

To confirm the integrity of the myofilament lattice structure from a functional perspective, we performed passive tension measurements with mechanically skinned fibers from H10–ECC and ISO under resting conditions. No appreciable differences in passive tension as a sign of connectin/titin degradation were found between ISO and H10–ECC, indicating that the sarcomeric serial elastic component connectin/titin remained functionally intact even in H10–ECC (Figure 6D). Electrophoretic analysis also confirmed that connectin/titin was intact even in H10–ECC (Figure 6E).

### 2.5. Alignment of Myoproteins

**Figure 7:**
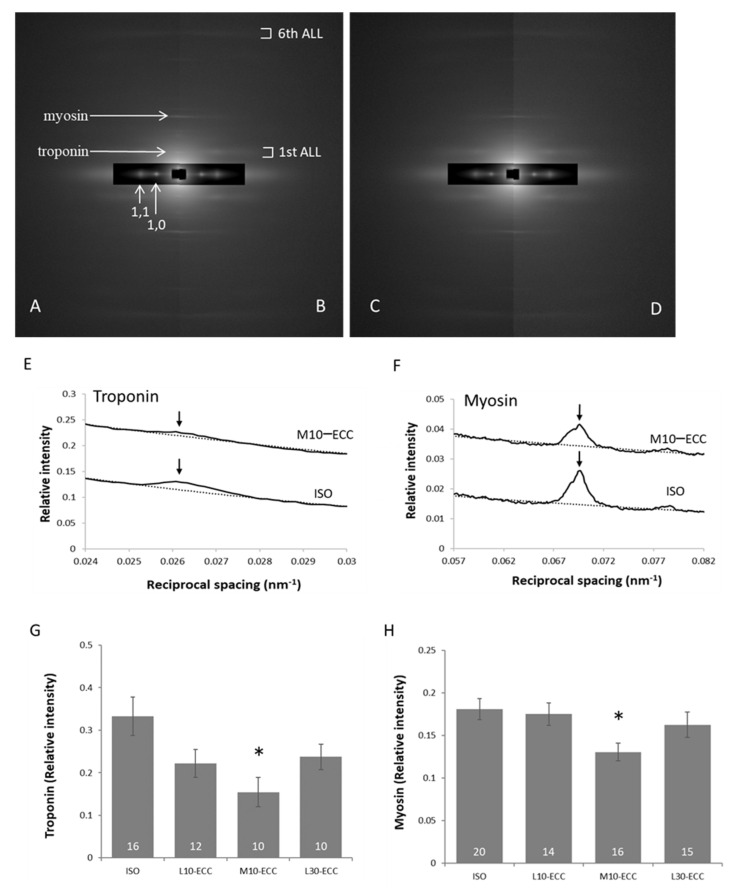
Analysis of rigor myofilaments. (**A–D**) Representative diffraction patterns of ISO (**A**), L10–ECC (**B**), M10–ECC (**C**), and L30–ECC (**D**) at the rigor condition. 1st ALL, 1st-order actin layer line; 6th ALL, 6th-order actin layer line; myosin, 3rd-order meridional myosin reflection at 1/14.4 nm^−1^; troponin, troponin reflection at 1/38 nm^−1^; 1,0 and 1,1, equatorial reflections. (**E**,**F**) Intensity profiles of troponin (**E**) and myosin (**F**) reflections along the meridian. The profile of M10–ECC was vertically shifted by 0.1 (**E**) and 0.02 (**F**) for visibility. Dotted line shows background. (**G**,**H**) Intensities of troponin and myosin reflections. Inside the bars are the numbers of samples. * *p* < 0.05 vs. ISO.

As stated earlier, meridional reflections and layer lines on the X-ray diffraction patterns were attenuated in H10–ECC. Therefore, detailed analyses were performed on the patterns of ISO, L10–ECC, M10–ECC, and L30–ECC. To focus on the molecular alignment of myofilaments, X-ray diffraction patterns of muscle specimens under the rigor conditions, where fluctuation of muscle proteins was reduced to enable precise analysis of the structure due to formation of the rigor cross-bridges, were analyzed (Figure 7). Slight but significant effects were found specifically in M10–ECC: intensity decrease in the thin-filament related meridional reflection of troponin at 1/38 nm^−1^ and the thick-filament related meridional reflection of myosin at 1/14.4 nm^−1^. Since L30–ECC showed no appreciable difference in these intensities compared with L10–ECC, the decrease in M10–ECC likely depended on instantaneous maximal tension rather than the tension-time integral.

## 3. Discussion

### 3.1. Muscle Damaging Effects

In this study, small but significant molecular traces of moderate intensity ECC were found within the sarcomere in M10–ECC. The trace depended on instantaneous maximal tension development rather than the total load of contraction and was more likely linked with the phosphorylation of MAPK and mTOR signaling proteins. On the other hand, the function (Figure 2) and fine molecular structure (Figure 6B) of muscle were significantly damaged by high-intensity contraction in H10–ECC, but well preserved against the largest tension-time integral of L30–ECC. This indicates that the muscle damaging effect of ECC depends on instantaneous tension rather than total contraction load, and tension development close to the maximal isometric tension level can be safely repeated without seriously damaging the muscle.

Instantaneously developed large tension in high-intensity ECC may cause damaging mechanical strain in force-generating and transducing systems such as the sarcomere, costamere, sarcolemma, fascia, and musculotendinous junctions. The neuromuscular junction on the sarcolemma may be affected by strain. The deterioration of extra-myofibrillar organelles in muscle cells may also impair muscle contractility through the malfunction of excitation-contraction coupling and metabolism. However, we consider that ECC would not directly damage extra-myofibrillar organelles because they should have tolerated abrupt physiological passive extension accompanying the concentric contraction of the antagonistic muscle in vivo. Since direct damage to the sarcoplasmic reticulum and mitochondria is expected to cause rapidly progressive deterioration through elevation of Ca^2+^ concentrations in muscle fibers [11], the steady time course of contractility change found in Figure 2B supports our view that extra-myofibrillar organelles would not be significantly damaged directly in the present ECC sessions. The steady time course also suggested that no secondary effect of experimental conditions, such as denervation, was seriously invoked within 1 h after the contraction sessions.

### 3.2. Muscle Strengthening Effect

Contractile activity strengthens skeletal muscle through anabolic growth, myogenic differentiation, and suppression of catabolism. It is generally considered that phosphorylated FoxO proteins are prevented from acting as an enhancer of muscle catabolism [12,13], phosphorylated MAPK proteins enhance myogenic differentiation [14], and phosphorylated proteins of the mTOR pathway accelerate anabolic muscle growth [15]. Among the signal proteins, we selected FoxO1, FoxO3, SAPK/JNK, p38MAPK, ERK, p70S6K, and 4E-BP1 as potential proteins that are expected to reflect the progress of signal transduction with phosphorylation cascades 1 h after the contraction sessions. Here, the slowly migrating γ-fraction of 4E-BP1 is known to represent the hyperphosphorylated form of the protein, which is fully effected with the phosphorylation at the serine 65 residue [16].

One of the selected MAPK proteins, p38MAPK, was reported to accelerate myogenetic differentiation as well as muscle catabolism through activation of the muscle atrophy F-box [17]. We presumed that this dual effect of p38MAPK would assist in the muscle remodeling necessary for muscle strengthening, at least at a relatively low contraction load.

As for the FoxO proteins, the total protein seemed to decrease after contraction sessions. The decrease in the short period suggested decomposition or transition of the proteins to an undetectable form in the muscle. In any event, the decrease in the total protein and concomitant increase in its phosphorylated form after the ECC sessions (Figure 3A,B) indicated a marked decrease in the unphosphorylated form of the FoxO proteins, which act as an enhancer of catabolic processes in muscle. Therefore, we used phosphorylation level, which is the ratio of the phosphorylated protein to total protein, as a conservative index of the effects of the contraction session on FoxO. The index of phosphorylation level was shared between the proteins of MAPK and mTOR pathways, where total protein showed no appreciable change after contraction sessions.

The correlation between phosphorylation levels of FoxO and the tension-time integral (Figure 3C,D), and phosphorylation levels of MAPK and mTOR pathways vs. maximal tension (Figure 4C,D and Figure 5B) ensured that the intensity range of the present ECC sessions was adequate for exploration of initial triggers for muscle strengthening effects of ECC near the threshold intensity.

Compared with the effects of ECC expected from the regression lines shown in Figure 3, isometric contraction of ISO had a smaller effect on FoxO1 and FoxO3 for their tension-time integral levels. This suggests that the present ECC sessions were more efficient than isometric contractions in suppressing muscle degeneration. Similarly, for MAPK and mTOR related signals, the present regression analyses in Figure 4 and Figure 5 indicated that ECC was more efficient in phosphorylating SAPK/JNK, p38MAPK, and 4E-BP1 for their maximal tension than isometric contraction. Therefore, we consider that the present ECC sessions were more efficient than isometric contraction sessions in activating muscle growth and myogenic differentiation. The specific efficiency of the present ECC sessions supports the involvement of ECC-specific triggering sites for muscle strengthening effects.

A significant correlation was found for the abundance of the S65 phospho-form of the γ-fraction in total 4E-BP1 with the maximal tension in the ECC group (Figure 5B). However, an earlier study reported that mTOR activation depends primarily on the tension-time integral independently of the contraction mode [18]. We consider that the difference could be ascribed to the shorter time period of the present contraction sessions with a far smaller tension-time integral compared with other studies.

Consistent with the present results, Martineau and Gardiner (2001) reported that ECC phosphorylated MAPK to a greater extent than isometric and concentric contractions [19]. On the other hand, Boppart et al. (2001) reported that extensive stretching of resting muscle by 20–25% also phosphorylated MAPK [20]. From the efficient phosphorylation of MAPK with only 10% stretch of the contracting muscle observed in the present study, we consider that the initial trigger for MAPK phosphorylation would be different between resting and contracting muscles.

### 3.3. Sarcomere Structure

The present X-ray analysis indicated that the lattice arrangement of myofilaments was well preserved, even with intense maximal contraction of H10–ECC. Since X-ray diffraction patterns were obtained from skinned fibers at resting conditions in the present study, the preserved 1,0 spacing of the thick filament lattice indicated a preserved force balance between neighboring myofilaments [21]. At resting conditions where myosin heads are detached from thin filaments, connectin/titin tethers neighbor thick filaments longitudinally and radially to resist the repulsive radial force acting between the filaments. Since intense strain in the sarcomere would not affect the radial repulsive force, which is generally considered to be electrostatic, the preserved lattice spacing would indicate that connectin/titin is functionally intact. The present passive tension measurement and electrophoretic analysis of H10–ECC confirmed that connectin/titin remained intact (Figure 6D,E). Therefore, we concluded that connectin/titin withstood the augmented force developed in the sarcomere during the high-intensity ECC. This is of interest because connectin/titin is suggested to stiffen during ECC, acting as a mechanosensor for muscle strengthening [22,23]. If connectin/titin bears a significant fraction of the augmented force during the present intense ECC without appreciable viscous relaxation, the connectin/titin of H10–ECC would have retained a history of intense strain.

We consider the decrease in the meridional troponin reflection at 1/38 nm^−1^ and myosin reflection at 1/14.4 nm^−1^ (Figure 7G,H) would represent a trace of intense local strain in thin and thick filaments, respectively. The present low and moderate ECC sessions that developed muscle tension comparable to isometric maximal tension could cause specifically intense local strain in myofilaments as follows. The stimulation frequency of low and moderate ECCs induced incomplete tetanus, where sparsely distributed contractile cross-bridges would be forced to extend, causing uneven intense strain along the myofilaments. Local strain in the extended portion of the myofilaments would likely exceed the maximal strain in more evenly strained myofilaments during isometric maximal contraction.

Meridional reflections on the X-ray diffraction patterns arise from the periodic repetition of mass along the longitudinal axis of the muscle fibers. Therefore, the decrease in intensity indicates the loss of mass or periodicity. Deviation of troponin from muscle cells was reported in humans 6–24 h after strenuous ECC [24,25]. Our electrophoretic study indicated that troponin content was preserved 1 h after the ECC sessions (unpublished). Complete or incomplete deviation of troponin from the regular sites on thin filaments within muscle fibers could be a possible cause for the intensity decrease at an earlier stage after the ECC sessions. Preserved intensity ratios of equatorial reflections at resting condition indicated that troponin functioned properly as a regulatory protein to prevent myosin heads from interacting with actin at the resting state, but its switching to contraction may be impaired, causing decreased maximal tension in M10–ECC (Figure 2B).

On the other hand, meridional myosin reflection is sensitive to the distribution and conformation of cross-bridges as well as the structure of the thick-filament backbone [26]. We can hardly distinguish the cause of the intensity decrease of the meridional myosin reflection at the present stage. However, the decrease represents a trace of ECC-specific strain on thick filaments as the decrease in the troponin reflection represents a trace of ECC-specific strain on thin filaments.

The correlation analyses indicated that the present ECC of low and moderate intensity was near the threshold level for the induction of muscle strengthening signals (Figure 3, Figure 4 and Figure 5). Therefore, we propose that the ECC-specific strain generated in thick and thin filaments is a probable candidate for triggering muscle strengthening effects that are specifically efficient in ECC. Since the X-ray diffraction patterns of L30–ECC showed no appreciable difference from those of L10–ECC, we consider that the ECC-specific strain depended on maximal tension rather than tension-time integral and would be more likely linked with ECC-specific activation of myogenic differentiation involving MAPK signals and anabolic muscle growth involving the mTOR pathway rather than the catabolic suppression involving FoxO signals.

### 3.4. Downstream of the Remodeling Process

In the present study, we tried to find traces of ECC-specific strain in sarcomeres after ECC sessions near the threshold intensity for the induction of muscle strengthening effects. We consider that the traces found in the present study represent potential candidates for mechanosensors that trigger downstream reactions that eventually lead to muscle strengthening.

Several extra-sarcomeric structures have recently been proposed as candidates for mechanosensors for the muscle strengthening effects of contraction. One of the proposed extra-sarcomeric candidates is costamere, which anchors Z discs to the extracellular matrix through the sarcolemma. Even a single passive stretch of muscle was reported to activate focal adhesion kinase (FAK) in costamere [27]. We consider that mechanosensors in extra-sarcomeric structures may work specifically in response to intense ECCs that develop large tension substantially exceeding the maximal isometric tension. On the other hand, for mild ECC, we postulated specific sites of initial action inside sarcomeres as reasoned earlier. This does not exclude the possibility that the postulated initial action in sarcomere secondarily affects some extra-sarcomeric structures, such as costamere, to evolve toward muscle strengthening.

### 3.5. Application to Rehabilitation Science

The present ECC sessions of low and moderate intensity affected the structure of sarcomere little, but was around the threshold levels for the induction of muscle strengthening effects. We provide a trustable guide for further exploration of mechanosensors for efficient muscle strengthening, which is an urgent issue in contemporary aged and super-aged societies requiring safe and effective rehabilitation for atrophic muscle disorders such as sarcopenia.

## 4. Materials and Methods

### 4.1. Animals

Animal experiments were reviewed and approved by the Institutional Animal Care and Use Committee of the Jikei University (No. 2015-122, Tokyo, Japan) and conformed to the Guidelines for the Proper Conduct of Animal Experiments of the Science Council of Japan (2006). Fifty-one F344 male rats, aged 7 weeks, were purchased from Sankyo Labo Service (Tokyo, Japan). All animals were housed for 1 week in an environment maintained at 22–24 °C with a 12–12 h light–dark cycle and received food and water ad libitum.

### 4.2. Solutions

Solution composition is listed in Table 2. All reagents were analytical grade.

### 4.3. In Situ Force Measurement

Each rat was anesthetized with isoflurane (3–5%) and maintained under anesthesia with intraperitoneal injection of triple anesthetic (0.15 mg/kg medetomidine + 2 mg/kg midazolam + 2.5 mg/kg butorphanol). Both hind limbs of each rat were utilized in successive series after covering the post-operative non-bleeding wound with an ordinary paper wiper. The rats were euthanized by pentobarbital sodium overdose at the end of the in vivo experiments.

A skin incision was made from the posterior surface of the lower leg, and the tissues covering the plantaris and tibial nerves were removed. The distal tendon of the plantaris muscle was cut and tied with a silk thread to the beam of a mechanical stimulation device (DPS-270; Dia Medical, Tokyo, Japan). The muscle was separated from the surrounding tissue to maintain muscle blood perfusion. The hip, knee, and ankle of the rat were fixed with clamps to an experimental chamber, in which whole hind limbs were immersed in Krebs-Ringer solution saturated with a mixed gas of 95% O_2_ and 5% CO_2_.

The distal portion of the tibial nerve was cut and placed on a pair of platinum wire electrodes in the solution. Rectangular pulses of 0.1 ms duration at supra-maximal intensity for the contraction (2–4 V) were applied through the electrodes connected to a stimulator (SEN 3301, Nihon Kohden, Tokyo, Japan).

The muscle force and displacement signals detected by the mechanical stimulation device were collected on a personal computer via an amplifier and an AD converter (NR-2000, Keyence, Osaka, Japan). Muscle cross-sectional area was estimated from muscle mass and length considering density and pennation angle to yield muscle tension (N·m^−2^) from the recorded force [28,29]. In each limb, we first determined the optimal length (L_0_) for maximal isometric twitch tension development.

To select a stimulation frequency for muscle activation, we examined contractile activation levels of muscle at 10–250 Hz with peak isometric tetanus tension at L_0_ using plantaris muscles in situ on both sides of four animals with characteristics similar to those of the main experiment. Based on these results, we selected frequencies of 100, 75, and 50 Hz for elicitation of high-, moderate-, and low-contractile activation levels, respectively (Figure 1A).

### 4.4. ECC Sessions

For bouts of ECC, we adopted 300 ms tetanus sessions repeated 10 times every 3 s. During each 300 ms session, the muscle was stretched from its preset length of 0.9 L_0_ to 1.0 L_0_ to obtain an ECC at stretching velocity of 0.33 L_0_·s^−1^. Immediately after each session, the muscle was released back to 0.9 L_0_ in 300 ms (Figure 1B).

The main experiment comprised six groups of randomly assigned plantaris muscles (Table 1). The H10–ECC, M10–ECC, and L10–ECC groups received 10 ECC sessions at high-, moderate-, and low-intensity stimulation at 100, 75, and 50 Hz, respectively. For comparison purposes, the ISO group received 10 sessions of isometric tetanus at 100 Hz at L_0_. The L30–ECC group received 30 low-intensity 50 Hz ECC sessions, and the control (CON) group received no contraction sessions.

Muscle contractility was evaluated before and 5, 30, and 60 min after the series of sessions with a single maximal isometric tetanus contraction of 300 ms duration at L_0_ (200–250 Hz). The plantaris muscle was dissected and weighed immediately after the last evaluation of contractility. Muscles for Western blotting and electrophoretic analyses were then rapidly frozen in isopentane, while others for X-ray diffraction and passive tension measurements were handled as follows. Fiber bundles of approximately 2 mm in diameter were carefully dissected from the muscle and treated with a surfactant (0.5% triton-X) in resting solution for 5 h to stop cellular processes with the removal of membrane systems and stored at −20 °C in resting solution diluted to half strength with glycerin.

### 4.5. Western Blotting

Signal proteins were analyzed using Western blotting. Each muscle specimen was minced and homogenized in cold lysis buffer. The homogenate was gently stirred for 60 min at 4 °C and then centrifuged at 16,000× *g* for 20 min at 4 °C. The supernatant was collected and protein content was determined using the Lowry method [30] with bovine serum albumin as a standard. The sample was solubilized in Laemmli sample buffer and boiled at 95 °C for 5 min. Using a 7.5–15% polyacrylamide gel, 20 µg of protein from each sample was separated by electrophoresis and subsequently transferred to a polyvinylidene difluoride membrane. After the transfer, the membrane was washed in Tris-buffered saline containing 0.1% Tween 20 (TBS-T) and blocked with 1% skim milk or 1% bovine serum albumin in TBS-T for 1 h at room temperature. After blocking, the membranes were washed and incubated overnight at 4 °C with primary antibodies for total FoxO1, FoxO1(S256), total FoxO3a, FoxO3a(Thr32), total SAPK/JNK, SAPK/JNK(Thr183/Tyr185), total p38MAPK, p38MAPK(Thr180/Tyr182), total p70S6K, p70S6K(T389), total 4E-BP1, 4E-BP1(S65), total ERK, and ERK(Thr202/Tyr204) (Cell Signaling Technology, Danvers, MA, USA). The membrane was then washed again in TBS-T and incubated for 1 h at room temperature with the appropriate secondary antibody (Cell Signaling Technology). A chemiluminescent reagent (ImmunoStar Zeta or LD, Wako Pure Chemical, Osaka, Japan) was used to detect protein bands. The bands were scanned using a chemiluminescence detector (LAS-3000 mini, Fujifilm, Tokyo, Japan), and their intensities were quantified using Multi Gauge (Fujifilm, Tokyo). The phosphorylation level of each protein was expressed as the ratio of phosphorylated protein to total protein on the band density basis. Since the bands were stained separately with respective primary antibodies, the level is not identical but proportional to the fraction of the phosphorylated form in the total protein.

### 4.6. X-Ray Diffraction Experiment

X-ray diffraction experiments were performed as described previously [31] at BL-6A of the Photon Factory (High Energy Accelerator Research Organization, Tsukuba, Japan). Specimens for X-ray diffraction experiments were prepared by carefully dissecting thin fiber bundles of approximately 0.2 mm diameter from the stored muscle bundles. Both ends of each specimen were tied to a stainless wire holder with silk monofilaments setting sarcomere spacing of the sample to be 2.5–3.2 μm with a help of laser diffraction. The specimen was placed in a small chamber equipped with two windows of Kapton (Dupont, DE, USA), which allowed an X-ray beam (0.15 nm wavelength, 0.7 mm wide, and 0.4 mm height) to pass through the specimen. The specimen was perfused with an appropriate solution at 20 °C. Diffraction patterns were recorded on an imaging plate set at 2.5 m downstream from the specimen and retrieved with BAS 2500 (Fujifilm, Tokyo) or Typhoon FLA 7000 (GE Healthcare, Buckinghamshire, UK). The typical X-ray exposure time required to obtain each diffraction pattern was 90 s. For detailed intensity analysis, the diffraction patterns from three different parts of each specimen were summed on a single imaging plate.

### 4.7. Analysis of X-Ray Diffraction Patterns

X-ray diffraction patterns were analyzed as previously described [31]. The four quadrants of the pattern were averaged. The reciprocal spacing was calibrated as the 3rd-order meridional myosin reflection at 1/14.3 nm^−1^ for the resting condition, and at 1/14.4 nm^−1^ for rigor condition. Reflections and layer lines were separated by assuming Gaussian distributions on an exponential background. The intensity along the equator in 0–0.013 nm^−1^ was integrated for the meridional troponin reflection at 1/38 nm^−1^ and for myosin reflection at 1/14.4 nm^−1^, and in 0–0.132 nm^−1^ for the 6th-order actin layer line (6th ALL) at 1/5.9 nm^−1^. For the equatorial reflections, the intensities within 0–0.013 nm^−1^ along the meridian were integrated. Since thin actin filaments are generally considered to be the most stable component in sarcomere [32], the intensities were normalized to that of the 6th ALL.

### 4.8. Passive Tension Measurement of Mechanically Skinned Fibers

A single muscle fiber was isolated from a stored muscle bundle, and the remnant of the sarcolemma was carefully peeled off with tweezers to obtain a mechanically skinned fiber. The fiber was then stretched in a resting solution while driving one end of the fiber with a manipulator (M-152, Narishige, Tokyo, Japan) in a step-wise manner and measuring passive tension with a capacitance tension transducer (403A, Cambridge Technology, Bedford, MA, USA) at the other end of the fiber. Cross-sectional images of the fiber observed with an in-house apparatus [33] were recorded with a digital camera (IXY Digital 920 IS, Canon, Tokyo, Japan) to measure the cross-sectional area.

### 4.9. Electrophoresis

Muscle specimens were boiled in SDS sample solution at 95 °C for 7 min and centrifuged (20 °C, 12,000× *g*, 15 min) to collect the supernatant. After adding half the volume of glycerol bromophenol blue to the collected supernatant, SDS-PAGE was performed using 12% polyacrylamide gel for troponin separation [34] and 2–6% gradient polyacrylamide gel for connectin/titin separation [35]. Density staining using CBB was read using a scanner (ChemiDoc Touch, Bio-Rad Laboratories, Hercules, CA, USA) for analysis, together with the supplied software or ImageJ [36].

### 4.10. Statistical Analysis

Experimental data are expressed as mean ± SEM. The results of X-ray diffraction and Western blotting were subjected to post hoc analysis by Tukey or Games–Howell when significant differences were found with ANOVA or Kruskal–Wallis test. The results of passive tension and electrophoresis were subjected to a two-sample *t*-test. Pearson was used for correlation analyses. The significance level was set at *p* < 0.05. All statistical analyses were performed with Modified R Commander 4.0.2 (Windows version; https://personal.hs.hirosaki-u.ac.jp/pteiki/research/stat/R/##1, accessed on 1 October 2020).

## 5. Conclusions

X-ray diffraction study together with parallel analyses of FoxO, MAPK, and mTOR related signals indicated that the present ECC sessions of low and moderate intensity had little effect on the structure of the sarcomere, but were near the threshold levels for the induction of muscle strengthening effects. Therefore, traces of mechanical strain were carefully searched in sarcomeres where ECC was expected to generate a specific local strain. We found slight but significant intensity decrease in meridional troponin reflection at 1/38 nm^−1^ and meridional myosin reflection at 1/14.4 nm^−1^ in the muscle after ECC sessions of moderate intensity. The decrease in troponin reflection indicates the effect of intense local strain on thin filaments, and a decrease in myosin reflection on thick filaments. Since repetition of the low-intensity ECC showed no appreciable effects on these reflections, ECC-specific effects would likely depend on instantaneous maximal tension rather than the total load of contraction.

## Figures and Tables

**Table 1:** Summary of animals and muscles.

	Muscle Group						
	CON	ISO	L10–ECC	M10–ECC	H10–ECC	L30–ECC	*p*
source animals							
*n*	13	12	13	13	12	11	
body weight (g)	157.5 ± 3.0	163.6 ± 4.1	163.1 ± 2.5	158.9 ± 3.3	164.0 ± 3.9	156.3 ± 2.3	n.s.
plantaris muscles							
*n*	13	12	13	13	12	13 *	
wet weight (mg)	147.4 ± 3.6	156.1 ± 5.2	154.6 ± 2.9	148.6 ± 3.4	154.4 ± 2.9	145.6 ± 3.0	n.s.
cross-sectional area (cm^2^)	0.115 ± 0.003	0.122 ± 0.004	0.121 ± 0.002	0.116 ± 0.003	0.123 ± 0.004	0.114 ± 0.002	n.s.
initial isometric tension (×10^−3^ N·m^−2^)	3.4 ± 0.1	3.3 ± 0.1	3.3 ± 0.1	3.4 ± 0.1	3.2 ± 0.1	3.4 ± 0.1	n.s.

* 4 muscles were from the same animals.

**Table 2:** Solution.

Krebs-Ringer solution	137 mM NaCl, 5 mM KCl, 1 mM MgCl_2_, 1 mM NaH_2_PO_4_, 1 g/L NaHCO_3_, 2 g/L Glucose, 2 mM CaCl_2_
lysis buffer	50 mM Tris-HCl (pH 7.5) ^i^, 1 mM EDTA ^i^, 1 mM EGTA ^i^, 0.5% (*w*/*v*) Sodium Deoxycholate ^ii^, 0.1% (*w*/*v*) SDS ^iii^, 150 mM NaCl, 1% (*v*/*v*) NP-40, 1 mM PMSF, 1 mM DTT, protease inhibitor cocktail ^iv^ phosphatase inhibitor cocktail ^v^
Laemmli sample buffer	62.5 mM Tris-HCl (pH6.8) ^i^, 0.05% (*w*/*v*) Bromophenol blue ^i^, 5% (*w*/*v*) SDS ^iii^, 50 mM DTT, 15% (*v*/*v*) Glycerol
SDS sample solution	100 mM Tris-HCl (pH 8.0) ^v^, 10% (*w*/*v*) SDS, 40 mM DTT, 5 mM EDTA ^i^
resting solution	5.1 mM Na_2_ATP ^ii^, 10 mM EGTA ^ii^, 5.6 mM Mg-(methanesulfonate)_2_ ^i, vi^, 75 mM K-(methanesulfonate) ^vi^^,^^vii^ 20 mM PIPES (pH 7.0) ^viii^, 10 mM creatine phosphate
rigor solution	130.5 mM K-(methanesulfonate) ^vi^^,^^vii^, 1.6 mM Mg-(methanesulphonate)_2_ ^i^^,^^vi^, 10 mM EGTA ^ii^ 20 mM PIPES (pH 7.0) ^viii^

^i^, Wako Pure Chemical, Tokyo, Japan, ^ii^, SIGMA, Kanagawa, Japan, ^iii^, Bio-Rad, California, USA, ^iv^, Roche Diagnostics, Basel, Switzerland, ^v^, Sigma Aldrich, MO, USA, ^vi^, Tokyo Chemical Industry, Tokyo, Japan, ^vii^, Kanto Chemical, Tokyo, Japan, ^viii^, Dojindo Molecular Technologies, Kumamoto, Japan, Others: Nacalai Tesque, Kyoto, Japan.

## Data Availability

All relevant data are contained within the article.
